# Highly Efficient Genome Engineering in *Bacillus anthracis* and *Bacillus cereus* Using the CRISPR/Cas9 System

**DOI:** 10.3389/fmicb.2019.01932

**Published:** 2019-08-27

**Authors:** Yanchun Wang, Dongshu Wang, Xiaojing Wang, Haoxia Tao, Erling Feng, Li Zhu, Chao Pan, Bowen Wang, Chunjie Liu, Xiankai Liu, Hengliang Wang

**Affiliations:** State Key Laboratory of Pathogens and Biosecurity, Beijing Institute of Biotechnology, Beijing, China

**Keywords:** CRISPR/Cas9, *Bacillus cereus* sensu lato group, *Bacillus anthracis*, *Bacillus cereus*, large genomic deletion, genomic site-specific mutagenesis

## Abstract

Genome editing is an effective tool for the functional examination of bacterial genes and for live attenuated vaccine construction. Here, we report a method to edit the genomic DNA of *Bacillus anthracis* and *Bacillus cereus* using the clustered regularly interspaced short palindromic repeats (CRISPR)/CRISPR-associated protein (Cas)9 system. Using two prophages in *B. anthracis* as targets, large-fragment deletion mutants were achieved with rates of 100 or 20%. In *B. cereus*, we successfully introduced precise point mutations into *plcR*, with phenotypic assays showing that the resulting mutants lost hemolytic and phospholipase enzyme activities similar to *B. anthracis*, which is a natural *plcR* mutant. Our study indicates that CRISPR/Cas9 is a powerful genetic tool for genome editing in the *Bacillus cereus* group, and can efficiently modify target genes without the need for residual foreign DNA such as antibiotic selection markers. This system could be developed for use in the generation of marker-free live anthrax vaccines or for safer construction of microbiological candidate-based recombinant *B. cereus*.

## Introduction

Targeted genome modification technology refers to the alteration of specific sites within the genome. Continuous research and development has so far established several theories and methods to achieve this goal (Joung and Sander, 2013; Gersbach et al., 2014; Maeder and Gersbach, 2016). Clustered regularly interspaced short palindromic repeats (CRISPR)/CRISPR-associated protein (Cas)9 technology is a recently developed targeted genome modification approach that has been successfully applied to a wide variety of eukaryotic and prokaryotic cells (Cong et al., 2013; Chandrasekaran et al., 2017). The technique uses a length of RNA to identify target sites, making it simpler to design and implement than other methods. It also offers the following unparalleled advantages: (1) a high modification efficiency that does not usually require screening markers, (2) the capacity to simultaneously modify multiple sites using different single guide (sg) RNAs, and (3) the ability to change the target sequence recognition site by altering only a short RNA sequence within the specific identification domain (Zhang et al., 2014; Chandrasekaran et al., 2017).

The *Bacillus cereus sensu lato* group consists of 18 closely related sporulating Gram-positive bacteria: *B. anthracis*, *B. cereus*, *B. thuringiensis*, *B. albus*, *B. cereus*, *B. cytotoxicus*, *B. luti*, *B. mobilis*, *B. mycoides*, *B. nitratireducens*, *B. pacificus*, *B. paramycoides*, *B. paranthracis*, *B. pseudomycoides*, *B. toyonensis*, *B. tropicus*, *B. wiedmannii*, and *B. weihenstephanensis* (Rossi et al., 2017; Acevedo et al., 2019). The main representatives of this group are *B. anthracis*, *B. cereus*, and *B. thuringiensis*. The chromosomes of these three bacteria show very high genetic similarity and their rRNA sequences are almost identical, differing only within the expected range of variation for a single species. However, the three species show highly divergent pathogenicity (Guinebretière et al., 2008; Liu et al., 2015; Okinaka and Keim, 2016). *B. thuringiensis* shows specificity for insect larvae, while *B. anthracis* and *B. cereus* are human pathogens. Although *B. anthracis*-induced anthrax is often fatal, *B. cereus* is a conditional pathogen that causes various diseases in humans such as periodontitis, food poisoning, and acute ophthalmitis (Kotiranta et al., 2000; Argôlo-Filho and Loguercio, 2014; Granum, 2017; Pilo and Frey, 2018).

The modification of target genes is important for research into the pathogenic mechanisms of bacterial strains. In recent years, many successful methods have been developed, however, most rely on the principle of homologous recombination (Dong and Zhang, 2014; Wang et al., 2016), and *B. anthracis*, *B. cereus*, and *B*. *thuringiensis* have very low homologous recombination efficiencies (Plaut and Stibitz, 2015). Additionally, research to date has mainly focused on the deletion of target genes, so precise point mutations and the removal of large fragments have not been achieved. Therefore, it is necessary to develop a new method to improve the efficiency of genetic modification in these bacteria. CRISPR/Cas9 technology has been widely used in *B. subtilis*, and a variety of relatively mature technical protocols have been constructed (Altenbuchner, 2016; Westbrook et al., 2016; Li et al., 2018). However, comparable research in the *Bacillus cereus* group is relatively limited. To date, the CRISPR/Cas9 system has only been used to construct *B*. *thuringiensis* mutants, and no related work has been reported in *B. anthracis* or *B. cereus* (Guo et al., 2019; Tan et al., 2019).

In the present study, we developed a highly efficient genome engineering protocol for *B. anthracis* and *B. cereus* based on the CRISPR/Cas9 system. Selecting two prophages contained within the genome of *B. anthracis* as targets, we successfully obtained large-fragment deletion mutants, resulting in a successful modification rate close to 20% for 50 kb fragment deletions. When the method was applied to delete a smaller genomic fragment from *B. anthracis*, a 100% modification rate was achieved. In *B. cereus*, we successfully introduced a precise point mutation into *plcR*, an important regulatory gene, and phenotype assays showed that mutants lost hemolytic and phospholipase enzyme activities. The protocol developed in this study will be a valuable tool for the generation of marker-free live anthrax vaccines and for the construction of safer microbiological candidate-based recombinant *B. cereus.*

## Materials and Methods

### Bacterial Strains and Growth Conditions

All bacterial strains used in this study and their relevant characteristics are listed in Table 1. Bacteria were grown aerobically at 30, 37, or 42°C. *Escherichia coli* strains were grown in Luria-Bertani (LB) broth and used as hosts for plasmid cloning. LB agar was also used for the selection of transformants. For competent cell preparation, *B. anthracis* strains were grown in brain heart infusion broth with the addition of 0.5% glycerol (BHIG; Becton, Dickinson and Company, Franklin Lakes, NJ, United States). Kanamycin was added to the growth media at a concentration of 50 μg/ml for *E. coli* and 25 μg/ml for both *B. anthracis* and *B. cereus*. To induce the expression of Cas9 protein, LB medium was supplemented with 25 μg/ml kanamycin and 0.4% mannose. Sheep blood agar plates and lecithin agar plates (LB agar supplemented with 1% (v/v) egg yolk) were used to detect hemolysis and phospholipase activity, respectively.

**TABLE 1:** Plasmids and strains used in this study.

**Plasmids and strains**	**Relevant characteristics**	**Source**
**Plasmids**		
pJOE8999	CRISPR-Cas9 vector; KanR	Altenbuchner, 2016
pJOE-Lam01	pJOE8999 with sgRNA-lam01 and homologous arms of lam01 from *B. anthracis* A16R, KanR	This study
pJOE-Lam03	pJOE8999 with sgRNA-lam03 and homologous arms of lam03 from *B. anthracis* A16R, KanR	This study
pJHRT	pJOE8999 with sgRNA-plcR and homologous arms of *plcR* from *B. cereus* HN001, KanR	This study
***B. cereus* group strains**		
*B. anthracis* A16R	pXO1^+^pXO2^–^, China vaccine strain	This laboratory
*B. anthracis* A16RΔ lam01	A16R excision prophage lambdaBa01	This study
*B. anthracis* A16RΔ lam03	A16R excision prophage lambdaBa03	This study
*B. anthracis* A16PI2	pXO1^+^pXO2^–^, deriving from A16 (pXO1^+^pXO2^+^)	Wang et al., 2011
*B. cereus* HN001	Wild-type *B. cereus*	This laboratory
*B. cereus* HN1M	*B. cereus* HN001 with G640T mutation in *plcR*	This study
***E. coli* strains**		
DH5α	Cloning strain	CWBIO, China
SCS110	*dam*–/*dcm*– strain used to produce unmethylated plasmid	Transgen, China

### DNA Manipulation

The preparation of plasmid DNA from *E. coli*, transformation of *E. coli*, and recombinant DNA techniques were carried out using standard procedures. *E. coli* DH5α competent cells were obtained from CWBio (Beijing, China), while *E. coli* SCS110 competent cells were purchased from Transgene (Beijing, China). Recombinant plasmid construction was carried out in *E. coli* DH5α. *B. anthracis* A16R chromosomal DNA was isolated using a Wizard Genomic Purification Kit (Promega, Madison, WI, United States) according to the protocol for the isolation of genomic DNA from Gram-positive bacteria. *B. anthracis* A16R and *B. cereus* HN001 were electroporated with unmethylated plasmid DNA isolated from *E. coli* SCS110, and electrocompetent cells were prepared as previously described (Wang et al., 2018).

### Plasmids Construction

All bacterial strains and plasmids used in this work are shown in Table 1, and PCR primers are listed in Table 2. Oligonucleotides for gRNA construction were designed using the online sgRNA design tool^1^. High-scoring 20-nucleotide (nt) sequences were selected.

**TABLE 2:** Primers used in this study.

**Name**	**Sequence (5′→3′)**	**Purpose**
Ulam03F	ACGCGTCGAC TTACGGCAATGTTCCAAAG	PCR of homology arms for lambdaBa03 excision
Ulam03R	TTTGGTCTC ATAATTTACTGACCGTATTGCTAAG	
Dlam03F	TTTGGTCTC AATTATCGTTTGATGTTATAAAAAG	
Dlam03R	GCTCTAGA CTCCAAACAAAGGTAAACTAGG	
sg-lam03F	tacgAACTAAGAAGGATATTCCAA	Target sequence for lambdaBa03 excision
sg-lam03R	aaacTTGGAATATCCTTCTTAGTT	
lam03p1	CCTGGGATTGATGATACGATGGC	PCR of lambdaBa03 excision mutant identification
lam03p2	TTGGTTTCGACGTAACTGACCAAG	
lam03p3	CCAAAATCAGCTGTAGCGATATTC	
lam03p4	TATCCATATAATGAGTTTTTTCTGCTTT	
lam03p5	CCTTCCTCGGCTTCTTCCATTG	
Ulam01F	ACGCGTCGAC TAATTGCAAATAACGG	PCR of homology arms for lambdaBa01 excision
Ulam01R	TTTGGTCTC AAGAGGGGATATATTCCGCACAC	
Dlam01F	TTTGGTCTC CCTCTTAGTAAAGAGAC	
Dlam01R	GCTCTAGA GGATTCTCAGATTTCAATC	
sg-lam01F	tacgTTAGACCCTCTACTACCAAG	Target sequence for lambdaBa01 excision
sg-lam01R	aaacCTTGGTAGTAGAGGGTCTAA	
lam01p1	TAAGCAATAATACATAGCAACAAACC	PCR of lambdaBa01 excision mutant identification
lam01p2	GTAATTTTCCCTTGGACAGCTG	
lam01p3	AAAGTGCAGCACCTACACTGAAAC	
lam01p4	AGTTTTCGATGAACTCAATGGCATG	
lam01p5	ATATTTTCAAAGAAATAAAAGCCC	
sg-plcRF	tacgAGGTGAATGCCTAGGGAAGT	Target sequence for site-specific mutagenesis of *plcR*
sg-plcRR	aaacACTTCCCTAGGCATTCACCT	
pJOEF	TAGTGTAGCCGTAGTTAGG	PCR identification of the introduction of pJHRT into HN001
pJOER	AAAGGGAATGAGAATAGTG	
plcRHMF	CTTCTGTTGATAAAGGGCAAAGAAG	PCR of *plcR* mutagenesis identification
plcRHMR	TTTAAAGTGATTGCAGAAGGTGTA	

To explore the feasibility of large chromosomal deletions using the CRISPR/Cas9 system in *B. anthrac*is, we selected the shortest (lambdaBa03, ∼16.8 kb) and longest (lambdaBa01, ∼50.5 kb) prophages in the *B. anthracis* genome as target fragments. *B. anthracis* A16R genomic DNA was used as a template to amplify upstream and downstream regions of lambdaBa03 using primers Ulam03F/Ulam03R and Dlam03F/Dlam03R, respectively, for vector construction. The two fragments were inserted into the corresponding *Sal*I and *Xba*I sites of pJOE899 (Altenbuchner, 2016). The resultant plasmid was then digested with *Bsa*I, and the large fragment (about 9 kb) of digested plasmid was ligated with the small double-stranded DNA,annealed with the two complementary oligonucleotides sg-lam03F/sg-lam03R, to obtain plasmid pJOE-Lam03. Plasmid pJOE-Lam01, used for the deletion of prophage lambdaBa01, was constructed in the same way.

To construct a vector for introducing a point mutation into *plcR*, a mutant *plcR* sequence containing a new termination codon, G640T, was synthesized by Genewiz (Suzhou, China). Five other same-sense mutation sites, C735G, A738T, G741C, G744A, and G747A, were also introduced into the synthesized sequence. This fragment was then cloned into the two *Sfi*I sites of plasmid pJOE8999. The resultant plasmid was digested by *Bsa*I, and the large fragment of digested plasmid was ligated with the small double-stranded DNA, annealed with the two complementary oligonucleotides sg-plcRF/sg-plcRR, to obtain plasmid pJHRT.

### Construction and Isolation of *B. anthracis* Mutants

The transformation and selection of *B. anthracis* A16R mutants was performed as described previously (Wang et al., 2018). The recombinant pJOE-based plasmids constructed above were introduced by electroporation into *B. anthracis* strain A16R. Transformants were selected at 30°C on BHIG medium containing kanamycin (25 μg/ml). Colonies were visible after 12–16 h. A single colony was then inoculated into liquid medium supplemented with 25 μg/ml kanamycin and incubated with shaking for 3 h at 37°C. Mannose (final concentration, 0.4%, w/v) was then added to induce the expression of the Cas9 protein. After a further 3 h of cultivation, serial dilutions of the culture were plated on LB agar containing 25 μg/ml kanamycin and 0.4% mannose and incubated at 37°C overnight. Following incubation, at least nine colonies were validated by PCR using multi-pair primers. *B. anthracis* A16R was used as a control. To eliminate the plasmid for genome editing, mutant strains were passaged up to three times in the absence of antibiotics to ensure plasmid loss, which was confirmed by the loss of kanamycin resistance.

### Construction and Isolation of *B. cereus* Mutants

Electrocompetent cells were prepared as described previously (Wang et al., 2018). Approximately 1 μg of plasmid pJHRT, derived from *E. coli* DH5α, was mixed with electrocompetent *B. cereus* cells and pulsed (0.6 kV, 500 Ω, and 25 μF) in a 0.1 cm gap cuvette. The cells were immediately resuspended in 1 ml of LB medium and incubated for 1 h at 30°C with shaking. Recovered cells were then spread on LB agar plates containing kanamycin (25 μg/ml).

Kanamycin-resistant *B. cereus* HN001 colonies were picked and assessed for the presence of recombinant plasmid pJHRT by PCR using the primer pair pJOEF/pJOER. Verified colonies were transferred to liquid medium supplemented with 25 μg/ml of kanamycin and incubated with shaking for 3 h at 30°C. Mannose (final concentration, 0.4%, w/v) was added to induce the expression of the Cas9 protein. Following incubation for a further 13 h at 28°C, the cultures were transferred to new liquid LB medium (1% dilution) and Cas9 expression was again induced. Finally, serial dilutions of the cultures were plated on sheep blood agar and incubated at 30°C overnight. Hemolysin activity was observed after 18 h, and clones with very small zones of clearing (indicating hemolysis) were selected for PCR-based analysis using primers plcRHMF/plcRHMR, followed by sequencing. Plasmids for genome editing were eliminated as described above for *B. anthracis*. Mutants were further identified by analysis of hemolysin and phospholipase activities as described above.

## Results

### Excision of Two Prophages in *B. anthracis*

**FIGURE 1:**
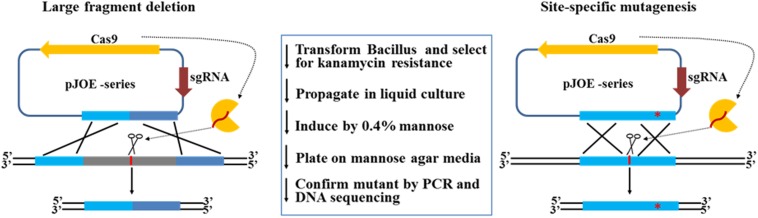
Schematic representation of genome engineering in *B. anthracis* and *B. cereus* via the CRISPR/Cas9 system. The all-in-one CRISPR-Cas9 plasmids based on pJOE consist of Cas9, sgRNA, and upstream and downstream homologous arms that serve as donor DNA. Transcription of the sgRNA allows the Cas9 protein to cleave at a specific site in the genome. The desired mutation is then achieved by recombination between donor DNA and the genome.

**FIGURE 2:**
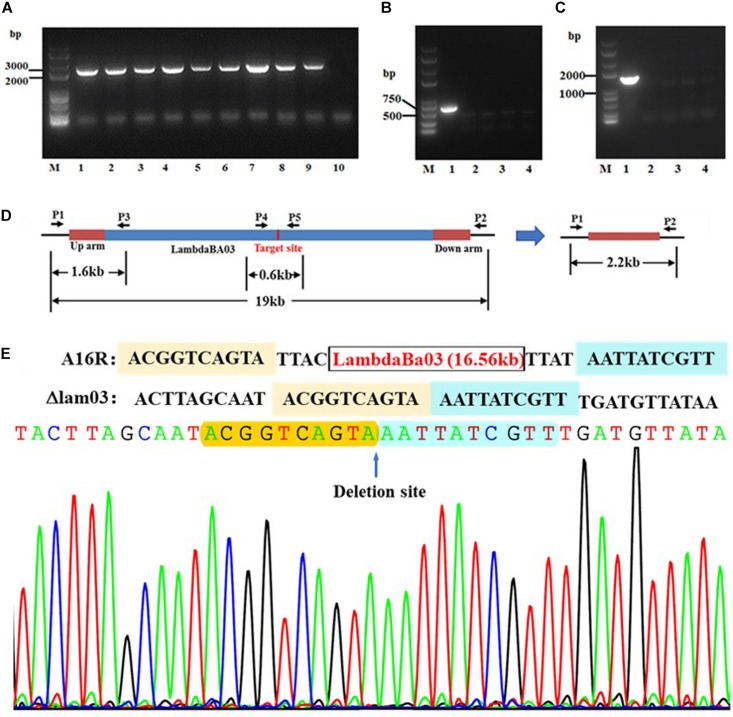
PCR-based verification of lambdaBa03 deletion in *B. anthracis* A16R. **(A)** Fragments amplified using primer pair lam03p1/lam03p2. The expected fragment in the mutant strain was approximately 2.2 kb (lanes 2–9). The fragment in the wild-type strain was > 19 kb, which exceeds the maximum amplification size under the PCR conditions used in this study (lane 1). **(B,C)** Fragments amplified using primer sets lam03p1/lam03p3 and lam03p4/lam03p5, respectively. Because of the location of the primers **(D)**, the expected fragments (1.6 or 0.6 kb, respectively) could only be amplified from the wild-type strain (lane 1) when the last two primer pairs were used for PCR (lane 1, A16R; lanes 2–4, mutant strains). **(E)** Sequencing data comparing wild-type strain A16R with the lambdaBa03 deletion mutant.

The schematic shown in Figure 1 outlines prophage excision and the method used to integrate donor DNA, sgRNA, and cas9 cassettes into a single plasmid. The target site for Cas9 was in the center of the lambdaBa03 prophage. Alternative mutants were detected by multiplex PCR using oligonucleotides lam03p1–lam03p5 (Figure 2). The region amplified by the lam03p1/lam03p2 primers in the control strain was > 19 kb in length, which exceeded the maximum amplification size under our PCR conditions. However, all randomly selected colonies (9/9) showed the expected 2.2 kb amplicon, indicating that the prophage locus had been deleted (Figure 2A). Genomic DNA was the extracted from four of the clones, and primers lam03p4/lam03p5 and lam03p1/lam03p3 were used for further PCR verification. No target sequences were amplified from the mutant strains, while amplicons were obtained from the control strain (Figures 2B,C). Because of the location of the primers (Figure 2D), the expected fragments (1.6 or 0.6 kb, respectively) could only be amplified from the wild-type strain and not from the mutants. In addition, DNA sequencing of the fragments amplified by lam03p1/lam03p2 primers confirmed that prophage lambdaBa03 was not present within that 2.2 kb amplicons (Figure 2E). Together, these results indicated that prophage lambdaBa03 had been completely eliminated in the mutant strains (Figures 2A–C,E).

**FIGURE 3:**
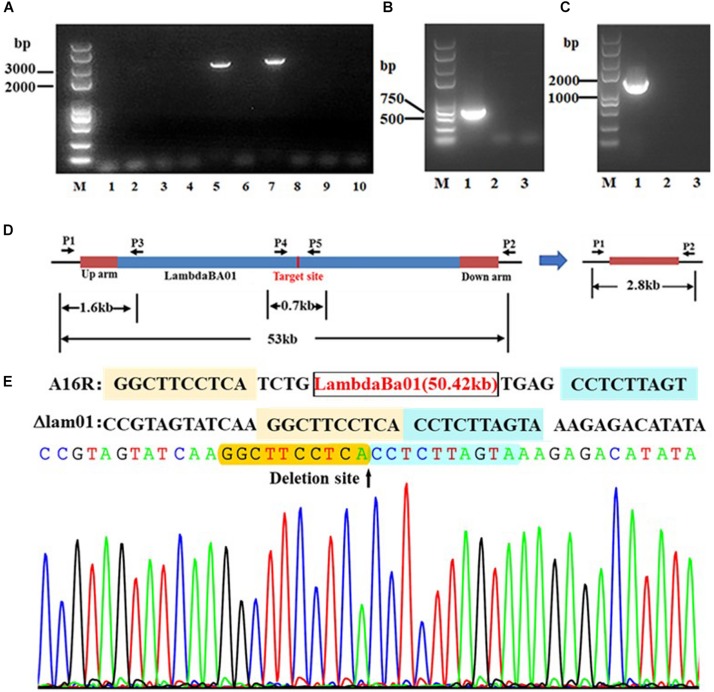
PCR verification of lambdaBa01 deletion in *B. anthracis* A16R. **(A)** Fragments amplified using primer pair lam01p1/lam01p2. The expected fragment in the mutant strain was approximately 2.8 kb (lanes 5, 7). **(B)** Fragments amplified using primer pairs lam01p1/lam01p3 and lam01p4/lam01p5. **(C)** Because of the location of the primers **(D)**, the expected fragments (1.6 or 0.7 kb, respectively) could only be amplified from the wild-type strain (lane 1) and not from the mutants (lanes 2 and 3). **(E)** Sequencing data comparing wild-type strain A16R with the lambdaBa01 deletion mutant.

Using a similar technique, we also excised the largest prophage, lambdaBa01, although with a relatively low success rate (2/9; Figures 3A–E). This efficiency was nevertheless higher than that achieved using other methods based on homologous recombination in *B. anthracis*.

### *plcR* Point Mutation in *B. cereus*

**FIGURE 4:**
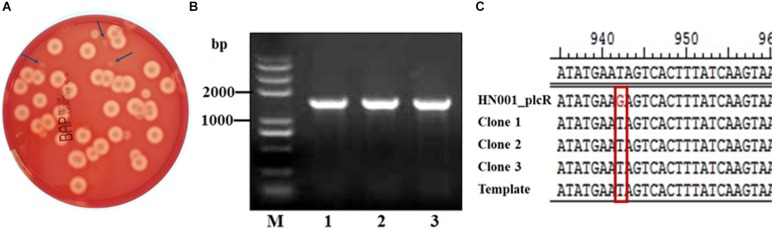
Verification of site-specific mutagenesis of *plcR* in *B. cereus* HN001. **(A)** Selection of positive mutants using hemolysin assays. Clones indicated by blue arrows are candidates with poor hemolytic activity. **(B)** PCR-amplified fragment of *plcR* for sequencing. **(C)** Sequence analysis of *plcR* from the putative mutant clones and the wild-type strain HN001. All three candidate clones contained the G640T point mutation.

**FIGURE 5:**
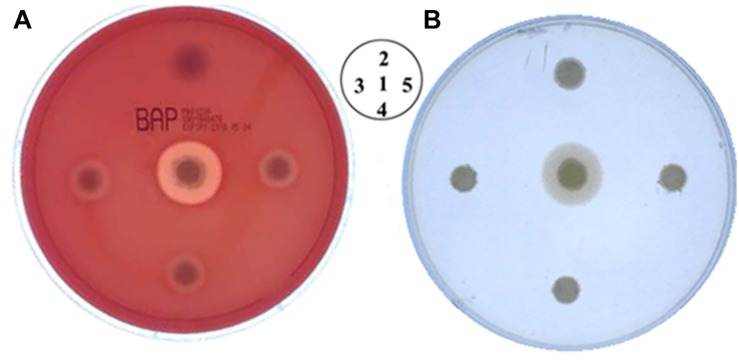
Phenotypic analysis of *B. cereus* HN1M. **(A)** Hemolytic activity assay. **(B)** Phospholipase activity assay. Similar to *B. anthracis*, the hemolytic enzyme activity of positive mutants was partially lost, while phospholipase activity was completely lost. Different strain locations are shown. 1. *B. cereus* HN001, 2. *B. anthracis* A16PI2, 3–5. *B. cereus* HN1M.

Using *B. cereus plcR* as a target, we investigated the feasibility of this method to carry out point mutations. We designed the G640T mutation based on the sequence of non-functional *B. anthracis plcR* gene, resulting in a non-functional PlcR protein and loss of hemolytic activity. As described above, a plasmid containing mutant *plcR* sgRNA and cas9 cassettes was electroporated into *B. cereus* strain HN001. Using this method, we successfully obtained recombinant strains that did not produce an obvious zone of clearing on sheep blood agar, indicating loss of hemolytic activity (Figure 4A). PCR-based analysis and DNA sequencing of these strains confirmed that the *plcR* gene contained the G640T mutation (Figures 4B,C). Further hemolysin and phospholipase activity assays revealed partial loss of hemolysin activity and total loss of phospholipase activity in the three selected mutant isolates, named *B. cereus* HN1M. The characteristics of the mutant strain were similar to those of *B. anthracis* strain A16PI2 (Wang et al., 2011), which contains a naturally-occurring nonsense mutation in *plcR* (Figure 5).

## Discussion

Deletion of important virulence-related genes is a means of constructing live attenuated vaccines, however, the choice of mutation method is pivotal to this process (Racz et al., 2013; Kamble and Lee, 2016). Desirable mutants are free from screening marker residues and should have a plaque-free presence within the genome (Mignon et al., 2015). To date, this has been achieved by various anti-selective technologies (Reyrat et al., 1998). We previously used a modified *pheS*^∗^ counter-selection mutant construction system to obtain a mutant strain with no selective markers (Wang et al., 2018). However, subsequent experiments showed that the removal of resistance markers using *cl-phe* led to unexpected mutations, as well as the deletion of large genomic fragments near the designated mutation site (Wang et al., unpublished data). These phenomena revealed the limitations of this method and the need to optimize the protocol or develop alternative techniques.

Although the CRISPR/Cas9 system has been used successfully in *Bacillus* species, especially *B. subtilis*, there are physiological differences between *B. anthracis* and *B. subtilis*. As such, many successful methods used in *B. subtilis* have not been implemented in *B. anthracis*. For example, single-stranded DNA oligonucleotide-mediated mutagenesis and PCR-based homologous recombination have been reported in many *B. subtilis* studies (Yan et al., 2008; Wang et al., 2012), but there are no corresponding reports in *B. anthracis*. Furthermore, our experiment also showed that the detail of execution process was not exactly the same. For example, the induction condition of Cas9 expression in *B. anthracis* and *B. cereus* was different. Therefore, it is imperative that we explore the feasibility of alternative systems in *B. anthracis* and *B. cereus*.

Our results confirm that the CRISPR/Cas9 system can be used to perform large-fragment deletions and precise point mutations in the genomes of *B. anthracis* and *B. cereus*, respectively. This work lays important foundations for the application of the method in related strains. Based on the current data and other published literature, there does not appear to be any significant difference in the success rate of the pJOE8999-based genome editing method between *B. subtilis* and *B. anthracis*. When the method was used to delete a large fragment (25 kb) in *B. subtilis*, >90% of the tested colonies showed the expected deletion (Altenbuchner, 2016). In the current study, large-fragment (∼17 kb) deletion mutants were achieved with a rate of 100%. Similarly, 100% (72/72) of tested colonies showed the correct sequence when this system was used to repair a 3 bp mutation of *trpC2* in *B. subtilis* in the same article. However, when the same system was used for site-specific mutagenesis of *plcR* gene in *B. cereus*, less than 10% (3/40) of clones contained the mutation, a mutation rate far lower than that regularly achieved in *B. subtilis*. Based on this finding, we thought the reason of this result is that PlcR is a fundamental regulatory protein in *B. anthracis* and likely plays an almost indispensable role in bacterial survival. To improve the efficiency of gene editing in *B. cereus* group strains, further optimization of the design of the gRNA, the mannose concentration, and the length of the homologous arms should be conducted.

The major shortcoming of the CRISPR/Cas9 system is the potential for off-target modification caused by repair of double-stranded chromosomal breaks through non-homologous end joining (NHEJ). Although a NHEJ system has been identified in *B. subtilis* (Weller et al., 2002; Moeller et al., 2011), published data indicate that the NHEJ system in *B. subtilis* is only active during sporulation, and therefore not active in growing cells (Moeller et al., 2011; Zheng et al., 2017). In addition, Toymentseva and Altenbuchner (2019) confirmed that the NHEJ system was not activated when CRISPR/Cas9-mediated genome editing was carried out in *B. subtilis*. No equivalent NHEJ system has been identified experimentally the *B. cereus* group strains investigated in the current study, although NHEJ-associated proteins (such as Ku-like proteins) have been identified by homology analysis. According to published research, NHEJ-mediated repair mechanisms in *B. anthracis* are inefficient compared with those found in *B. subtilis* (Cote et al., 2018). Therefore, strains with non-specific cleavage might not survive in the absence of homologous arms to repair the genomic DNA breaks. This characteristic would ensure that almost all of the resulting clone contained the correct modification. Nevertheless, future studies should carry out genome-wide sequencing of the strains to analyze possible off-target effects and determine the reliability of the system.

Using *B. anthracis*, we eliminated two different prophages using CRISPR/Cas9 technology, confirming that this system is a meaningful genetic manipulation tool for research in this important human pathogen. Although deletion of large fragments of the *B. anthracis* genome has been reported, the protocol used was very complex (Pomerantsev et al., 2006). To accomplish deletion, genes on either side of the target fragment were deleted by means of homologous recombination and the Cre/LoxP system, and *loxP* sites were introduced at both ends of the target fragment. Recombination at the *loxP* sites by Cre recombinase resulted in deletion of the large intervening region. However, the *loxP* sites remain in the genome, which may affect the long-term stability of the bacterial genome. Although such genetic instability can be minimized using mutational *lox* sites (*lox66* and *lox71*), scars (*lox72*) still remain in the target genome (Lambert et al., 2007). Overall, our method is far simpler than the Cre/LoxP method, and the correspondingly shorter experimental period is valuable for mutant construction.

We also successfully introduced a G640T point mutation into *plcR* in *B. cereus*. This technique could be used to study the specific regulatory mechanisms of important virulence-related genes in *B. cereus*. In most *Bacillus* strains, the products of *plcR* and its related genes have important physiological functions. Yet in *B. anthracis*, the most virulent of the *B. cereus* group species, PlcR is non-functional (Mignot et al., 2001; Gohar et al., 2008; Bhunia, 2018). The mutant strain developed in the current study could therefore be used as a possible model strain to explore why *B. anthracis* containing a nonsense mutation at position 640 forms a stop codon that fails to produce a functional PlcR protein.

Therefore, we have developed a simple and fast genetic manipulation tool for the study of *B. anthracis*. As well as being a method for label-free deletion of large fragments in *B. anthracis*, providing a new and effective technique for constructing live attenuated vaccine strains, this technique also allows us to obtain recombinant strains with precise deletions of the prophages. This could provide the necessary model strains for functional study of genes carried by these prophages in the growth, reproduction, and pathogenic process of *B. anthracis*. These studies would ultimately help to reveal the biological importance of the *B. anthracis*-specific prophages.

## Data Availability

The raw data supporting the conclusions of this manuscript will be made available by the authors, without undue reservation, to any qualified researcher.

## Author Contributions

YW, XL, and CL designed the research. YW, DW, XW, HT, EF, LZ, CP, and BW performed all experiments. CL and HW analyzed data. YW, XL, and HW wrote the manuscript. All authors reviewed the final manuscript.

## Conflict of Interest Statement

The authors declare that the research was conducted in the absence of any commercial or financial relationships that could be construed as a potential conflict of interest.

## Footnotes

1 https://sg.idtdna.com/site/order/designtool/index/CRISPR_CUSTOM
